# Does health differ between participants and non-participants in the MRI-HUNT study, a population based neuroimaging study? The Nord-Trøndelag health studies 1984–2009

**DOI:** 10.1186/1471-2342-12-23

**Published:** 2012-07-30

**Authors:** Lasse-Marius Honningsvåg, Mattias Linde, Asta Håberg, Lars Jacob Stovner, Knut Hagen

**Affiliations:** 1 Department of Neuroscience, Norwegian University of Science and Technology, Trondheim, 7491, Norway; 2 Norwegian National Headache Centre, St. Olavs University Hospital, Trondheim, Norway

**Keywords:** General population study, Population characteristics, Participation rates, Cardiovascular disease, BMI, Cholesterol, Education, Neuroimaging, Magnetic resonance imaging

## Abstract

**Background:**

Bias with regard to participation in epidemiological studies can have a large impact on the generalizability of results. Our aim was to investigate the direction and magnitude of potential bias by comparing health-related factors among participants and non-participants in a MRI-study based on HUNT, a large Norwegian health survey.

**Methods:**

Of 14,033 individuals aged 50–65, who had participated in all three large public health surveys within the Norwegian county of Nord-Trøndelag (HUNT 1, 2 and 3), 1,560 who lived within 45 minutes of travel from the city of Levanger were invited to a MRI study (MRI-HUNT). The sample of participants in MRI-HUNT (n = 1,006) were compared with those who were invited but did not participate (n = 554) and with those who were eligible but not invited (n = 12,473), using univariate analyses and logistic regression analyses adjusting for age and education level.

**Results:**

Self-reported health did not differ between the three groups, but participants had a higher education level and were somewhat younger than the two other groups. In the adjusted multivariate analyses, obesity was consistently less prevalent among participants. Significant differences in blood pressure and cholesterol were also found.

**Conclusion:**

This is the first large population-based study comparing participants and non-participants in an MRI study with regard to general health. The groups were not widely different, but participants had a higher level of education, and were less likely to be obese and have hypertension, and were slightly younger than non-participants. The observed differences between participants and non-invited individuals are probably partly explained by the inclusion criterion that participants had to live within 45 minutes of transport to where the MRI examination took place. One will expect that the participants have somewhat less brain morphological changes related to cardiovascular risk factors than the general population. Such consequences underline the crucial importance of evaluation of non-participants in MRI studies.

## Background

In recent years, participation rates in epidemiological studies have been declining [1]. However, very few population-based and clinical studies have extensive health-related information regarding non-participants. A systematic review of 116 articles published in 2009 in a specified epidemiological journal showed that demographic analyses on participants versus non-participants were performed in only 10% [2]. However, such data are of major importance because validity and generalizability of findings are limited if participants differ substantially from non-participants. Indeed, several earlier epidemiological studies have found that non-participants tend to have lower health status than participants [3-5].

The Nord-Trøndelag Health Study (HUNT) is a large scale epidemiological study conducted in three waves in the period 1984 to 2008, and included evaluation of non-participants [6-9]. Magnetic resonance imaging of the brain was one of many sub-studies integrated into the last of these (MRI HUNT). The aim of the present study is to compare the health related factors collected in the period 1984 to 2008 between participants and non-participants in MRI HUNT, which can aid in the interpretation of future reports based on MRI HUNT. To the best of our knowledge, extensive description of non-participants in a population-based MRI study has not been done earlier.

## Methods

The HUNT studies were conducted during 1984 to 1986 (HUNT 1), 1995 to 1997 (HUNT 2) and 2006 to 2008 (HUNT3) in the Norwegian county of Nord-Trøndelag, which is one of 19 counties in Norway, and fairly representative of the rest of the country. In all three surveys, the entire population aged 20 years or older was invited to participate. The first questionnaire (Q1) was enclosed with the invitation letter. All were invited to a brief clinical consultation including measurement of blood pressure (BP), height and weight. In HUNT 2 and 3, blood samples were also acquired. Not all participants took part in all elements of the health examination, answered all the questions or filled out additional questionnaires. In all three HUNT-studies, women were more likely to attend, and the participation was highest in the age group 50–79, with lower participation for those older and younger [10].

In HUNT 1, the main topics were hypertension, diabetes mellitus, lung diseases and health-related quality of life [11]. Of 85,100 eligible individuals, 74,977 (88%) answered Q1 and also participated in the medical examination. Previously published studies about the HUNT 1 population has shown that the main reasons for not attending were that they were busy, lacked interest, had moved or had health problems [6], and that, among the elderly, non-participants had poorer health than participants [12].

HUNT 2 was a more comprehensive study, covering a wide range of topics, described elsewhere [7].The Q1 included several demographic variables, such as marital status, education, working status, exercise, use of tobacco, alcohol, and caffeine, and anxiety and depression. Out of 92,936 invited individuals, 66,140 (71%) participated. A study of a random sample of non-participants, showed that the main reasons for non-participation in the age group 20–69 were lack of time, having moved out of the county, being too busy at work, having forgotten the invitation, or no particular reason, whereas among those ≥70 many did not feel the need to attend the health survey [7]. Of the participants in HUNT 2, 47,286 had also participated in HUNT 1.

Apart from a few minor modifications (adding or removing certain items), HUNT 3 is equivalent to HUNT 2 concerning the health-related topics. Of 94,194 invited adults, a total of 50,839 (54%) answered the Q1 and attended the medical examination. A participation study showed that those who were employed, earned a high salary, had a higher level of education or lived in an inland area were more likely to participate [13]. Overall, a total of 27,980 subjects have participated in all three HUNT-studies.

### MRI-HUNT study (performed 2007–2009)

The cohort invited to participate in the MRI-HUNT study was drawn from the population who had participated in HUNT 1, 2 and 3 and was between 50 and 65 years at the time of the MRI acquisition (n = 14,033). The exclusion criteria were limited to MRI contraindications; pacemaker of the heart, clipped cerebral aneurysm, cochlear implants, severe claustrophobia or weight above 150 kg. Furthermore, individuals were only included if their travelling distance to the location of the MRI examination at Levanger hospital did not exceed 45 minutes. The aim was to achieve 1000 participants. To attain this, 1,560 individuals who fulfilled these criteria were selected for potential participation. Those aged 50–65 years who had participated in HUNT 1, 2 and 3, but were not invited, were defined as non-invited (MRI-ni) (n = 12,473). MRI contrast agents were not used, and the invitation letter informed that the session would last approximately 30 minutes.

A selective invitation was made in order to obtain the desired sex and age distribution within the group. Because of this stratification process, 66 of the 1,560 persons fulfilling inclusion criteria were not invited to the examination, leaving 1,494 who were invited to attend the study. 1,088 (73%) of these invited individuals gave informed consent and 1,006 (476 males and 530 females) (67% of invited) had successful MRI examinations and were defined as MRI participants (MRI-p). A total of 488 persons were invited, but did not participate, mostly because they declined the invitation or did not answer (n = 406). Other reasons for not participating were that the scanning was terminated due to claustrophobia (n = 16), muscle cramps (n = 5) or the image acquisition was unsuccessful due to metallic artifacts (n = 3). Some also cancelled the session prior to the scanning (n = 28), did not show up (n = 5), had contraindications (n = 4),moved (n = 1), died (n = 1), were above 65 years (n = 1) or were hospitalized (n = 1). Data collection was closed when the number of participants had passed 1,000, and the planned scanning of 17 individuals was consequently cancelled.

**Figure 1:**
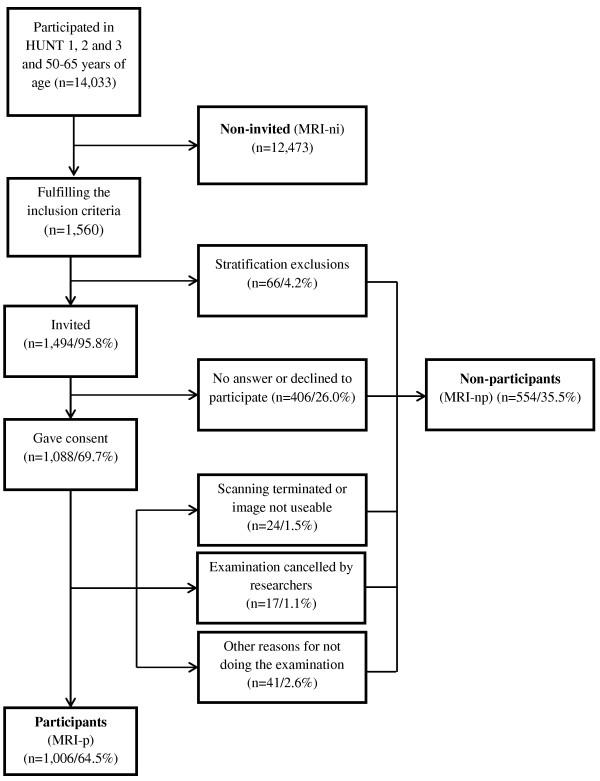
Flowchart describing the cohort.

Even though the actual number of individuals that were invited but did not participate was 488, the de-identified data file we received from HUNT research center included the 66 persons who were excluded due to stratification. Thus, this group of MRI non-participants (MRI-np) consisted of a total of 554 persons. The numerous reasons for non-participation and ineligibility are summarized in a flowchart (Figure 1).

### Variables

For the purpose of this article, divorce and separation were recoded into the same marital status category. In HUNT 1 and 2, education (originally five levels) was categorized in two levels (≤ 12 years or >12 years). In HUNT 3, education level was measured using information from HUNT 2. As to employment status, the question differed slightly in the three HUNT studies. In order to enable meaningful comparisons between them, we differentiated only between employed and non-employed.

Based on measurement at the medical examinations in HUNT 1, 2 and 3, the proportion of obese (BMI >30 kg/m^2^) was calculated, and systolic and diastolic BP was registered. In HUNT 2 and 3 blood samples were analyzed for HDL-cholesterol, total cholesterol, non-fasting glucose, and triglycerides.

Subjective health was in the three HUNT surveys assessed with the question “How is your health at the moment?”, and the four response categories ranging from “poor” to “very good”, was merged to two. HUNT 2 and 3 contained identical screening questions on headache (“Have you suffered from headache during the last 12 months?”) and chronic musculoskeletal complaints (Have you suffered from pain or stiffness in muscle and joints lasting for at least 3 months during the last year?”). HUNT 2 and 3 had a series of questions regarding mental health which constituted the Hospital Anxiety and Depression Scale (HADS).

HUNT 1, 2 and 3 included quite similar, but not identical, questions regarding alcohol, physical activity and smoking habits. In order to enable meaningful comparisons between the surveys, individuals were divided into two groups with regard to use of alcohol (abstainers versus non-abstainers), physical activity (active versus inactive), and smoking (current smokers versus others).

There were very few missing data on demographical variables and measured data. No imputation was done for missing data on measured variables. The questions which we based two of our variables on (non-employment in HUNT 2 and daily smoking in HUNT 3) were asked in such a manner that missing data most sensibly was interpreted as a negative answer.

### Ethics

The Norwegian Data Inspectorate, the Norwegian Board of Health, and the Regional Committee for ethics in Medical Research had approved all HUNT studies, including MRI-HUNT, and the regional committee also approved the present analysis. All participants in HUNT 1, 2, 3 and MRI-HUNT gave their informed, written consent.

### Statistical methods

Differences between MRI-p, MRI-np and MRI-ni were analyzed using data from all three HUNT-studies and men and women were analyzed separately. In the univariate analyses, Chi-squared test was used for categorical data, and one-way ANOVA for continuous data. If a Bonferroni-adjusted p-value <0.001 (0.05/50) was achieved, multivariate analyses were performed for both sexes in all three HUNT-studies, using logistic regression with odds ratio (OR) and 95% confidence intervals (CI), adjusting for age (continuous variable), and education level (five categories). Total cholesterol, HDL-cholesterol, non-fasting glucose, HADS-A (Anxiety) and –D (Depression) were dichotomized using 75-percentile as cutoff. Additionally, in order to examine how robust the findings were, the multivariate analyses were repeated including only individuals weighing <120 kg. Data analysis was performed with the Predictive Analytic SoftWare (PASW) Statistics v17.0 by SPSS Inc., IBM Company (Chicago, IL, USA).

## Results

Unadjusted analyses evaluating differences between MRI-p, MRI-np, and MRI-ni for HUNT 1, 2 and 3 are shown in Table 1, 2 and 3. Participants were slightly younger and a larger proportion had higher level of education compared to the two other groups. Consequently, adjustments for these two factors were performed in the multivariate analyses.

**Table 1:** HUNT 1: Characteristics of participants in the MRI study (MRI-p), non-participants (MRI-np) and non-invited (MRI-ni)

**VARIABLES**	**WOMEN**				**MEN**			
	***MRI-p***	***MRI-np***	***MRI-ni***	***p***	***MRI-p***	***MRI-np***	***MRI-ni***	***p***
**Demographic**	***(n = 530)***	***(n = 286)***	***(n = 6678)***		***(n = 476)***	***(n = 268)***	***(n = 5695)***	
Age (mean [SD])	35.1 [4.2]	35.1 [4.3]	35.6 [4.5]	0.007^1^	35.3 [4.1]	35.2 [4.2]	35.7 [4.5]	0.07^1^
Education >12 years (n [%])	124 [27.3]	58 [24.2]	779 [13.9]	<0.001^2^	117 [28.1]	57 [27.3]	882 [18.6]	<0.001^2^
Separated or divorced (n [%])	24 [4.5]	10 [3.5]	303 [4.5]	0.72^2^	10 [2.1]	5 [1.9]	161 [2.8]	0.43^2^
Non-employed (n [%])	16 [3.0]	8 [2.8]	339 [5.0]	0.03^2^	13 [2.7]	5 [1.9]	196 [3.4]	0.28^2^
**Health-related**								
Fair or poor health (n [%])	64 [12.1]	28 [9.8]	834 [12.3]	0.45^2^	43 [9.1]	28 [10.5]	619 [10.9]	0.46^2^
Physical inactivity (n [%])	23 [5.0]	14 [5.7]	387 [6.8)]	0.25^2^	31 [7.2]	16 [7.5]	412 [8.6]	0.56^2^
Daily smoking (n [%])	159 [34.3]	84 [34.1]	2108 [37.4]	0.25^2^	119 [27.7]	74 [34.7]	1574 [32.7]	0.08^2^
Alcohol abstainers(n [%])	29 [6.3]	19 [7.8]	29 [6.3]	0.65^2^	13 [3.0]	9 [4.2]	163 [3.4]	0.73^2^
BMI (mean [SD])	22.6 [2.7]	23.5 [3.3]	23.4 [3.4]	<0.001^1^	24.6 [2.3]	24.9 [2.9]	24.8 [2.8]	0.128^1^
BMI ≥30 kg/m^2^ (n [%])	8 [1.5]	19 [6.6]	339 [5.1]	<0.001^2^	9 [1.9]	18 [6.7]	267 [4.7]	0.004^2^
**Laboratory measurements**								
SBP (mean [SD])	119.3 [13.0]	121.0 [12.5]	121.0 [13.4]	0.019^1^	129.1 [12.6]	129.9 [12.8]	130.6 [12.8]	0.052^1^
SBP ≥140 mmHg (n [%])	34 [6.4]	24 [8.4]	603 [9.0]	0.13^2^	93 [19.5]	52 [19.4]	1276 [22.7]	0.14^2^
DBP (mean [SD])	78.6 [8.7]	79.7 [9.3]	77.9 [9.6]	0.003^1^	83.8 [8.6]	84.2 [9.9]	82.7 [9.3]	0.003^1^
DBP ≥90 mmHg (n [%])	55 [10.4]	48 [16.8]	759 [11.3]	0.013^2^	119 [25.0]	76 [28.4]	1253 [22.3]	0.033^2^

1. One-way analysis-of-variance 2. Chi-squared test.

BMI: Body mass index; SBP: Systolic blood pressure; DBP: Diastolic blood pressure; Fair or poor health: Fair or poor self-perceived health.

**Table 2:** HUNT 2: Characteristics of participants in the MRI study (MRI-p), non-participants (MRI-np) and non-invited (MRI-ni)

**VARIABLES**	**WOMEN**				**MEN**			
	***MRI-p***	***MRI-np***	***MRI-ni***	***p***	***MRI-p***	***MRI-np***	***MRI-ni***	***p***
**Demographic**	***(n = 530)***	***(n = 286)***	***(n = 6678)***		***(n = 476)***	***(n = 268)***	***(n = 5695)***	
Age (mean [SD])	45.7 [4.3]	45.8 [4.3]	47.1 [4.5]	<0.001^1^	46.0 [4.2]	45.9 [4.2]	47.2 [4.5]	<0.001^1^
Education >12 years (n [%])	179 [34.0]	94 [33.2]	1349 [20.1]	<0.001^2^	156 [32.9]	79 [29.8]	1292 [22.9]	<0.001^2^
Separated or divorced (n [%])	72 [13.6]	29 [10.1]	713 [10.5]	0.083	41 [8.6]	32 [11.9]	449 [7.9]	0.055^2^
Non-employed (n [%])	14 [2.6]	8 [2.8]	220 [3.2]	0.659^2^	7 [1.5]	7 [2.6]	131 [2.3]	0.463^2^
**Health-related**								
Fair or poor health (n [%])	105 [19.8]	60 [21.1]	1607 [23.9]	0.063^2^	84 [17.8]	36 [13.5]	1062 [18.8]	0.087^2^
Physical inactivity (n [%])	18 [3.8]	11 [4.3]	308[5.0]	0.361	22 [5.4]	14 [6.2]	389 [7.8]	0.151
Daily smoking (n [%])	159 [32.6]	88 [33.6]	2321 [36.1]	0.216^2^	119 [26.9]	82 [32.5]	1568 [28.7]	0.288^2^
Alcohol abstainers(n [%])	38 [7.3]	28 [9.9]	548 [8.2]	0.426^2^	19 [4.0]	15 [5.8]	290 [5.2]	0.498^2^
BMI (mean [SD])	25.1 [3.4]	26.3 [4.3]	26.0 [4.2]	<0.001^1^	26.3 [2.7]	26.7 [3.4]	26.7 [3.2]	0.038^1^
BMI ≥30 kg/m^2^ (n [%])	46 [8.7]	47 [16.4]	1039 [15.4]	<0.001^2^	44 [9.3]	43 [16.0]	827 [14.6]	0.004^2^
Headache(n [%])	274 [58.9]	130 [53.9]	3167 [54.4]	0.162^2^	156 [36.4]	73 [34.6]	1644 [35.4]	0.883^2^
Musculoskeletal pain(n [%])	281 [53.4]	137 [48.2]	3654 [54]	0.159^2^	222 [46.8]	113 [42.3]	2618 [46.1]	0.444^2^
HADS A score(mean [SD])	4.2 [3.4]	4.6 [3.5]	4.6 [3.4]	0.030^1^	3.7 [2.9]	3.8 [2.8]	4.0 [3.1]	0.061^1^
HADS D score(mean [SD])	2.9 [2.8]	2.9 [2.9]	3.4 [2.9]	<0.001^1^	3.5 [2.9]	3.3 [2.6]	3.7 [3.0]	0.026^1^
Antihypertensive use (n [%])	14 [2.6]	13 [4.5]	370 [5.5]	0.017^2^	17 [3.6]	9 [3.4]	321 [5.6]	0.052^2^
**Laboratory measurements**								
SBP (mean [SD])	126.1 [15.6]	128.1 [16.6]	130.2 [17.7]	<0.001^1^	133.2 [13.0]	135.1 [15.3]	135.9 [15.1]	0.001^1^
SBP ≥140 mmHg (n [%])	83 [15.7]	71 [24.8]	1760 [26.0]	<0.001^2^	134 [28.2]	89 [33.3]	2046 [36.0]	0.002^2^
DBP (mean [SD])	77.6 [10.0]	78.7 [10.6]	78.9 [10.7]	0.022^1^	82.0 [9.1]	83.6 [10.7]	83.3 [10.2]	0.034^1^
DBP ≥90 mmHg (n [%])	67 [12.6]	43 [15.0]	1038 [15.3]	0.248^2^	94 [19.8]	64 [24.0]	1417 [25.0]	0.041^2^
Cholesterol (mean [SD])	5.6 [1.0]	5.6 [1.0]	5.8 [1.1]	<0.001^1^	5.9 [1.0]	5.8 [1.1]	6.0 [1.1]	0.001^1^
High cholesterol (n [%])	99 [18.7]	52 [18.2]	1578 [23.3]	0.008^2^	122 [25.7]	59 [22.1]	1682 [29.6]	0.008^2^
HDL Cholesterol(mean [SD])	1.5 [0.4]	1.5 [0.4]	1.5 [0.4]	0.048^1^	1.2 [0.3]	1.2 [0.3]	1.3 [0.3]	<0.001^1^
Triglycerides (mean [SD])	1.3 [0.8]	1.4 [0.8]	1.4 [0.9]	0.008^1^	2.1 [1.2]	2.1 [1.3]	2.1 [1.3]	0.831^1^
Glucose (mean [SD])	5.1 [0.8]	5.3 [1.6]	5.2 [0.9]	0.005^1^	5.4 [1.2]	5.4 [1.1]	5.4 [1.3]	0.558^1^
Glucose ≥5.6 mmol/l (n [%])	106 [20.0]	74 [25.9]	1742 [25.8]	0.013^2^	146 [30.7]	83 [31.1]	1824 [32.1]	0.783^2^

1. One-way analysis-of-variance 2. Chi-squared test.

BMI: Body mass index; SBP: Systolic blood pressure; DBP: Diastolic blood pressure; Fair or poor health: Fair or poor self-perceived health; Antihypertensive use: Current use of antihypertensive medication; Glucose: Non-fasting glucose; High Cholesterol: Cholesterol ≥6.6 mmol/l.

**Table 3:** HUNT 3: Characteristics of participants in the MRI study (MRI-p), non-participants (MRI-np) and non-invited (MRI-ni)

**VARIABLES**	**WOMEN**				**MEN**			
	***MRI-p***	***MRI-np***	***MRI-ni***	***p***	***MRI-p***	***MRI-np***	***MRI-ni***	***p***
**Demographic**	***(n = 530)***	***(n = 286)***	***(n = 6678)***		***(n = 476)***	***(n = 268)***	***(n = 5695)***	
Age (mean [SD])	57.6 [4.2]	57.6 [4.3]	58.2 [4.5]	<0.001^2^	57.9 [4.1]	57.7 [4.2]	58.3 [4.5]	0.027^1^
Education >12 years (n [%])	179 [34.0]	94 [33.2]	1349 [20.1]	<0.001^2^	156 [32.9]	79 [29.8]	1292 [22.9]	<0.001^2^
Separated or divorced (n [%])	86 [16.2]	42 [14.7]	881 [13.0]	0.085^2^	56 [11.8]	41 [15.3]	619 [10.6]	0.071^2^
Non-employed (n [%])	101 [19.3]	79 [27.9]	1889 [28.1]	<0.001^2^	76 [16.0]	42 [15.7]	1233 [21.7]	0.001^2^
**Health-related**								
Fair or poor health (n [%])	164 [31.7]	100 [35.8]	2097 [32.2]	0.418^2^	110 [23.4]	70 [26.6]	1459 [26.3]	0.389^2^
Physical inactivity (n [%])	11 [2.1]	10 [3.5]	184 [2.8]	0.473^2^	18 [3.8]	11 [4.1]	283 [5.0]	0.407^2^
Daily smoking (n [%])	92 [17.4]	60 [21.0]	1661 [24.5]	0.001^2^	70 [14.7]	57 [21.3]	1046 [18.4]	0.058^2^
Alcohol abstainers(n [%])	18 [3.4]	14 [4.9]	263 [4.0]	0.579^2^	9 [1.9]	5 [1.9]	123 [2.2]	0.873^2^
BMI (mean [SD])	26.6 [4.1]	27.8 [4.9]	27.5 [4.6]	<0.001^1^	27.3 [3.1]	27.9 [3.9]	27.9 [3.6]	0.003^1^
BMI ≥30 kg/m^2^ (n [%])	110 [20.8]	79 [27.6]	1761 [26.0]	0.022^2^	93 [19.5]	69 [25.7]	1413 [24.9]	0.030^2^
Headache(n [%])	192 [42.5]	79 [36.1]	2177 [38.2]	0.155^2^	120 [29.1]	60 [29.4]	1298 [27.9]	0.792^2^
Musculoskeletal pain(n [%])	286 [63.4]	141 [64.7]	3576 [62.3]	0.718^2^	200 [47.8]	108 [52.4]	2351 [50.4]	0.493^2^
HADS A score(mean [SD])	4.0 [3.5]	4.5 [3.5]	4.3 [3.5]	0.158^1^	3.2 [2.8]	3.6 [3.1]	3.5 [3.1]	0.254^1^
HADS D score(mean [SD])	2.9 [2.6]	3.0 [2.6]	3.3 [2.9]	0.002^1^	3.4 [2.9]	3.7 [2.9]	3.7 [2.9]	0.243^1^
Antihypertensive use (n [%])	101 [19.1]	54 [18.9]	1638 [24.2]	0.005^2^	93 [19.5]	60 [22.4]	1495 [26.3]	0.003^2^
**Laboratory measurements**								
SBP (mean [SD])	129.7 [17.1]	132.0 [19.3]	132.2 [18.7]	0.006^1^	134.2 [16.5]	136.0 [17.8]	135.4 [17.0]	0.277^1^
SBP ≥140 mmHg (n [%])	136 [25.8]	100 [35.7]	1880 [32.2]	0.004^2^	165 [35.2]	96 [36.1]	1805 [36.7]	0.792^2^
DBP (mean [SD])	73.2 [10.5]	74.1 [10.9]	73.5 [10.5]	0.485^1^	79.9 [9.9]	80.9 [10.0]	79.9 [10.2]	0.278^1^
DBP ≥90 mmHg (n [%])	34 [6.4]	21 [7.5]	387 [6.6]	0.830^2^	71 [15.1]	43 [16.2]	798 [16.2]	0.816^2^
Cholesterol (mean [SD])	5.9 [1.1]	5.8 [1.1]	6.0 [1.1]	0.055^1^	5.5 [1.0]	5.4 [1.0]	5.6 [1.0]	0.001^1^
High cholesterol (n [%])	149 [30.1]	79 [30.0]	2120 [31.9]	0.589^2^	75 [16.5]	33 [13.3]	1152 [20.5]	0.003^2^
HDL Cholesterol(mean [SD])	1.5 [0.4]	1.5 [0.4]	1.5 [0.4]	0.364^1^	1.2 [0.3]	1.2 [0.3]	1.2 [0.3]	0.103^1^
Triglycerides (mean [SD])	1.5 [0.8]	1.6 [0.9]	1.6 [0.9]	0.134^1^	1.8 [1.0]	1.9 [1.2]	1.9 [1.2]	0.055^1^
Glucose (mean [SD])	5.4 [1.5]	5.6 [1.4]	5.6 [1.4]	0.018^1^	5.8 [1.8]	5.7 [1.6]	6.0 [1.8]	0.039^1^
Glucose ≥5.6 mmol/l (n [%])	76 [15.4]	48 [18.3]	1387 [20.9]	0.009^2^	125 [27.5]	60 [24.1]	1763 [31.3]	0.016^2^

1. One-way analysis-of-variance 2. Chi-squared test.

BMI: Body mass index; SBP: Systolic blood pressure; DBP: Diastolic blood pressure; Fair or poor health: Fair or poor self-perceived health; Antihypertensive use: Current use of antihypertensive medication; Glucose: Non-fasting glucose; High Cholesterol: Cholesterol ≥6.6 mmol/l.

In univariate analyses, no differences were found for self-reported health. The most consistent differences between the groups were found for BP and BMI. In all three HUNT studies, participants were less likely to be obese than the two other groups, and in the second study, participants had lower mean systolic BP. Significant but less consistent differences were also found for cholesterol, HADS-D and employment status. These variables were therefore examined further with multivariate analyses.

In the multivariate analyses, adjusting for differences in age and education level, obesity were less common among participants in all studies, most evident in HUNT 1 (Table 4). Excluding those weighing >120 kg did not change the results (data not shown). Regarding BP, the most consistent findings were among women, in whom systolic BP ≥140 mm Hg was less common among MRI-p than among the two other groups in HUNT 2 and 3. Among men, these differences were only found between MRI-p and MRI-ni in HUNT 2. Also among men in HUNT 2, HDL-cholesterol ≥1.6 was less common among MRI-p than among MRI-ni. Furthermore, cholesterol >6.5 and HDL-cholesterol >1.7 were less common among MRI-p compared to MRI-ni in HUNT 3. Regarding employment status and HADS, the picture was less clear. Among women in HUNT 3, HADS-D score was lower for MRI-p compared to MRI-ni and a smaller proportion of MRI-p was unemployed compared to MRI-np.

**Table 4:** Odds ratio (OR) with 95% confidence interval (CI) for participants (MRI-p), non-participants (MRI-np) and non-invited (MRI-ni) related to various health-related variables and adjusted for age and education

**VARIABLES**	**WOMEN**			**MEN**		
	***MRI-p***	***MRI-np***	***MRI-ni***	***MRI-p***	***MRI-np***	***MRI-ni***
**HUNT 1**	**OR**	**OR (95% CI)**	**OR (95% CI)**	**OR**	**OR (95% CI)**	**OR (95% CI)**
BMI (≥30 kg/m^2^ vs. <30 kg/m^2^)	1.00	3.38 (1.39-8.21)**	2.53 (1.24-5.16)*	1.00	4.97 (1.89-13.07)**	2.86 (1.26-6.50)*
SBP (≥140 mmHg vs. <140 mmHg)	1.00	1.48 (0.82-2.70)	1.25 (0.84-1.86)	1.00	0.87 (0.57-1.35)	1.15 (0.89-1.49)
Non-employed (yes vs. no)	1.00	1.05 (0.41-2.71)	1.59 (0.88-2.87)	1.00	0.48 (0.13-1.73)	1.06 (0.57-1.98)
**HUNT 2**						
BMI (≥30 kg/m^2^ vs. <30 kg/m^2^)	1.00	2.07 (1.34-3.21)**	1.63 (1.19-2.23)**	1.00	1.78 (1.12-2.82)*	1.54 (1.11-2.13)**
SBP (≥140 vs. <140 mmHg)	1.00	1.82 (1.26-2.62)**	1.52 (1.19-1.95)**	1.00	1.27 (0.91-1.76)	1.30 (1.06-1.61)*
Total Cholesterol (≥6.6 vs. <6.6)	1.00	0.98 (0.67-1.43)	1.05 (0.83-1.32)	1.00	0.82 (0.57-1.17)	1.12 (0.90-1.39)
HADS D score (≥5.0 vs. <5.0)	1.00	0.98 (0.69-1.39)	1.21 (0.98-1.50)	1.00	0.83 (0.59-1.17)	1.02 (0.83-1.26)
Non-employed (yes vs. missing)	1.00	1.05 (0.43-2.55)	1.11 (0.64-1.93)	1.00	1.76 (0.61-5.10)	1.38 (0.64-2.99)
HDL-Cholesterol (≥1.6 vs. <1.6)	1.00	0.76 (0.56-1.02)	0.99 (0.82-1.18)	1.00	1.19 (0.76-1.88)	1.51 (1.13-2.03)**
**HUNT 3**						
BMI (≥30 kg/m^2^ vs. <30 kg/m^2^)	1.00	1.45 (1.04-2.04)*	1.21 (0.97-1.51)	1.00	1.37 (0.96-1.96)	1.30 (1.03-1.65) *
SBP (≥140 vs. <140 mmHg)	1.00	1.64 (1.19-2.26)**	1.25 (1.02-1.54)*	1.00	1.08 (0.79-1.49)	1.05 (0.86-1.29)
Total Cholesterol (≥6.5 vs. <6.5)	1.00	1.02 (0.73-1.41)	1.07 (0.88-1.31)	1.00	0.79 (0.51-1.23)	1.32 (1.02-1.71)*
HADS D score (≥5.0 vs. <5.0)	1.00	1.12 (0.77-1.63)	1.30 (1.04-1.63)*	1.00	1.28 (0.90-1.83)	1.14 (0.92-1.42)
Non-employed (yes vs. no)	1.00	1.73 (1.19-2.51)**	1.27 (0.99-1.62)	1.00	0.90 (0.57-1.42)	1.16 (0.88-1.54)
HDL-Cholesterol (≥1.7 vs. <1.7)	1.00	0.87 (0.63-1.20)	0.93 (0.77-1.14)	1.00	1.20 (0.63-2.29)	1.69 (1.12-2.56)*

*p <0.05 ** P <0.01.

BMI: Body mass index; SBP: Systolic blood pressure.

## Discussion

To the best of our knowledge this paper is the first extensive description of non-participants in a population-based MRI study. The present study demonstrate that participants volunteering and successfully completing MRI scanning were not widely different from those who did not participate, and self-reported health did not differ between them. Notably, however, participants were less often obese, had a higher level of education, and were somewhat younger than MRI-np and MRI-ni. Risk factors for cardiovascular disease (high BP and cholesterol) were less prevalent among participants. The participants were also more likely to be employed. Additionally, HADS-score was found to be lower among participants, indication less psychological symptoms. It appears that most of the differences were present at all survey points. However, cholesterol and HADS-score were only available from the last two surveys, therefore, it is difficult to ascertain differences between the groups with regard to these factors were present from the first study or had developed over time.

The main objective of this study of non-participants was to enable a careful evaluation of the generalizability of results from future MRI-HUNT analyzes, which has rarely been possible in previous population-based MR-studies [2]. Strengths of the study were the large number of participants, the population-based design, and the long follow-up (>20 years) with three data points for each participant.

A limitation of the study was that some questions were not filled out by every participant, but the problem with missing data was not extensive on each question, and was unlikely to influence results. It should be noted that 66 out of 1560 eligible and selected candidates were not invited due to stratification, but we had to count them as invited, because it was impossible to trace them in the de-identified data file. We cannot see that this could markedly influence the results. One may also note that all three groups consisted of individuals who had participated in all three HUNT-studies and therefore might be more compliant than the rest of the population. Also, multiple comparisons increase the risk of type I error. To avoid false positive results, a Bonferroni-adjusted p-value of 0.001 was chosen for the univariate analyses.

Participation rates have declined in HUNT 1, 2 and 3(88%, 71% and 54%). Such decline in epidemiological studies seems to be a general tendency in later years [1]. Therefore, it has become increasingly important to analyze characteristics of non-participants. However, evaluation of non-participants in MRI studies is in general lacking, and if done, is mostly restricted to demographic variables [14]. A Finnish study examining non-participation rates among patients with psychiatric illnesses suggested that subjects with psychosis were less likely to participate in an MRI-study [15]. Similarly, in the present study participating women had lower HADS-D than non-participating, possibly indicating a lower burden of psychiatric illness. One may speculate whether subjects with higher level of anxiety or depression tend to avoid MRI for fear of the investigation itself, or of the result.

There is a considerable decline in participation rates from the first to the last HUNT study. It seems that the reasons for not participating were quite similar in HUNT 1 and 2, but with some differences. In both, being busy and having moved were the main reasons, but having health problems were specific for HUNT 1, and forgetting the invitation and not having the desire to participate, were only reported in HUNT 2. Self-reported reasons for not participating are not available in HUNT 3. Thus it is not possible to ascertain whether the reasons for not participating differed from those in the first two studies.

There were slight, but significant, differences with regard to clinical characteristics and presence of risk factors between the three groups. This finding shows the need to take into account differences in risk factor profiles at baseline in participants versus non-participants in future population based MRI studies. Importantly, power might be weakened due to lower prevalence of people with risk factors in the study population. This will, however, not have any effect on associations or risk analyses. One of the exclusion criteria was weight >150 kg, but this probably does not explain the lower BMI among participants, since only one individual was above this weight in HUNT 3.

However, place of living within Nord-Trøndelag is a factor that probably accounts for part of the difference. In the MRI-HUNT study, the participants had to live <45 minutes of travel from the town where the scans were performed (Levanger), due to budget restraints, and to increase participation. In all three HUNT-studies, a higher proportion with obesity and lower education levels has been found in rural communities [8], and the higher BMI may also explain the higher BP and cholesterol among MRI-ni.

This cannot explain differences between MRI-np and MRI-p, because both groups lived in the same area. The lower level of education and increased BMI and BP among MRI-np may be explained by generally lower participation rates among individuals with lower education and poorer health [16]. Conceivably, higher BMI among MRI-np compared to MRI-p may also be a result of overweight people, even those well below 150 kg, tend to refrain from participation in fear of being too big for the scanner. Different proportions of obese individuals might further have contributed to differences in other health related measures (cholesterol and BP).

Cardiovascular risk factors (like obesity and hypertension) are related to a risk of stroke and TIA, and also to alterations in brain morphometry [17-25].Lower participation rates among those with high cardiovascular risk could therefore lead to an underestimation of vascular brain changes in the general population. The prevalence of these changes in the MRI-p will therefore most probably represent the minima, and to some extent one can correct for the bias. In other population-based MRI-studies, various types of bias may be present, probably related to the mode of recruitment and a host of other factors, but their direction and magnitude are largely unknown.

## Conclusions

Self-reported health did not differ between participants, non-participants and non-invited, but, participants had higher education level, lower BMI, lower BP, and were somewhat younger. The observed differences between participants and non-invited individuals are probably partly explained by the inclusion criterion that participants had to live within 45 minutes of transport to the hospital where the MRI examination took place. Since increased BMI and BP impact brain morphometry, this should be taken into consideration and preferably corrected for when the results of the MRI-HUNT will be published. Because the generalizability of results may be influenced by selective participation, we recommend that non-participation studies in MRI research should be mandatory.

## Competing interests

The authors declare that they have no competing interests.

## Authors’ contributions

LMH contributed to designing the study, analyzing the data, interpreting the results and writing and revising the article. ML contributed to designing the study, interpreting the results and revising the article. AH contributed to collecting the data, designing the study, interpreting the results and revising the article. LJS was leader of the MRI-HUNT-study, and contributed to designing the study, interpreting the results and revising the article. KH contributed to designing the study, applying for data access, analyzing the data, interpreting the results and writing and revising the article. All authors have read and approved the final manuscript.

## Pre-publication history

The pre-publication history for this paper can be accessed here:

http://www.biomedcentral.com/1471-2342/12/23/prepub
